# Morphological and Molecular Characterizations of *Cochliomyia hominivorax* (Diptera: Calliphoridae) Larvae Responsible for Wound Myiasis in French Guiana

**DOI:** 10.3390/diagnostics13152575

**Published:** 2023-08-02

**Authors:** Mohammad Akhoundi, Alexandre Mathieu, Wajih Hannachi, Jade Nasrallah, Guillaume Quezel, Romain Blaizot, Denis Blanchet, Habib Ben Romdhane, Loïc Epelboin, Arezki Izri

**Affiliations:** 1 Parasitology-Mycology Department, Avicenne Hospital, AP-HP, Sorbonne Paris Nord University, 93000 Bobigny, France; 2 Groupe d’Étude et de Protection des Oiseaux en Guyane, Rémire-Montjoly 97354, French Guiana; 3 ENT Department, Centre Hospitalier de Cayenne Andrée Rosemon, Cayenne 97306, French Guiana; 4 Infectious and Tropical Diseases Unit, Centre Hospitalier de Cayenne Andrée Rosemon, Cayenne 97306, French Guiana; 5 Department of Dermatology, Centre Hospitalier de Cayenne Andrée Rosemon, Cayenne 97306, French Guiana; 6 Parasitology Mycology Universitary Laboratory, Centre Hospitalier de Cayenne Andrée Rosemon, Cayenne 97306, French Guiana; 7 Radiology Department, Avicenne Hospital, AP-HP, 93000 Bobigny, France; 8 Centre d’Investigation Clinique Antilles Guyane CIC Inserm 1424, Centre Hospitalier de Cayenne Andrée Rosemon, Cayenne 97306, French Guiana; 9 Unité des Virus Émergents (UVE: Aix-Marseille Université-IRD 190-Inserm 1207-IHU Méditerranée Infection), 13005 Marseille, France

**Keywords:** diptera flies, *Cochliomyia hominivorax*, larvae, wound myiasis, pre-urban areas, immunocompetent patient, French Guiana

## Abstract

Myiasis is an ectoparasitic infection caused by the larvae of true flies (Diptera). We came across a rather rare case of myiasis in an immunocompetent 34-year-old man from French Guiana with advanced wound myiasis masquerading as cavitary myiasis and a history of cholesteatoma surgery in the left ear. The Diptera larvae responsible for the disease were isolated and identified using morphological and molecular approaches as *Cochliomyia hominivorax.* We underline the importance of this parasitosis as the second case of myiasis caused by *C. hominivorax* and the first case of wound myiasis in this overseas department of France and its incidence in pre-urban areas of the capital, Cayenne, in South America.

## 1. Introduction

Myiasis is an ectoparasitic disease of human or animal tissues caused by the development of larvae (maggots) of various fly species within the Diptera order [1]. It generally invades vertebrates but occasionally occurs in humans [2]. Human myiasis is a neglected and under-reported disease [3], mainly due to its negative social implications. The infection is more prevalent in tropical and subtropical regions of the world because the warm and humid climatic conditions provide suitable breeding places for flies [4,5,6]. Furthermore, it is more common in rural regions than in urban areas, especially in developing countries with a lack of basic sanitation, close contact with livestock animals, and inadequate garbage disposal [6,7]. Men are more infected than women, mainly due to outdoor activities and less personal hygiene [8]. It generally affects people of low socioeconomic level, immunocompromised, or those with psychiatric disorders [6,9].

The Diptera order is composed of the Nematocera and Brachycera suborders. The latter comprises several important genera responsible for myiasis in humans in the Old World and New World, including *Dermatobia*, *Calliphoridae*, *Cochliomyia*, *Cordylobia*, *Chrysomyia*, *Cuterebra*, *Oestrus, Gasterophilus*, *Hypoderma*, *Phaenicia* (known as *Lucilia*), *Sarcophaga*, and *Wohlfahrtia* [10,11].

Myiasis may be classified in different ways. Based on the affected body location, it can be classified as (i) cutaneous, (ii) oral, (iii) nasal, (iv) cerebral, (v) ocular, (vi) intestinal, and (vii) urogenital myiasis [12,13]. According to the host–parasite relationship, it can be subdivided into obligatory, facultative, or accidental myiasis [12]. On the other hand, it can be considered furunculoid (penetration of the dipteran larva into healthy skin), migratory, cavity, or wound myiasis [14]. Except for cutaneous myiasis, all other aforementioned myiases are leading causes of hospitalization, amputation, reduced mobility, loss of social inclusion, and poor quality of life [15]. 

There are a variety of predisposing factors, depending on the type of myiasis. Wound myiasis is commonly linked to socioeconomic factors, while cavity myiasis is associated with the presence of animals, and ultimately furunculoid myiasis is related to exposure to mosquitoes or larval-infected tissues (or flies, mango trees, etc.). However, low socioeconomic status, malnutrition, drug use, alcoholism, low oral hygiene, facial trauma, open wounds, mouth-breathing, mental retardation, psychological disorders, advanced age, poor hygiene conditions, vascular disorders, necrosis, immunocompromised conditions, diabetes mellitus, and living in close contact with domestic animals are conditions that favor human myiasis [16]. The severity of myiasis is associated with infested body location and clinical manifestations (e.g., lesions and tissue inflammation); therefore, it requires early diagnosis and intervention. 

The diagnosis of myiasis can be challenging, mostly due to numerous fly species associated with infestation, and multiple clinical presentations and symptom variations based on infected-body location. Sometimes, the symptoms are similar to those of other infectious diseases, such as cellulitis, impetigo, herpes zoster, or leishmaniasis [17,18]. Therefore, misdiagnosis is not uncommon. Accurate and prompt diagnosis is important, not only to relieve the patient’s symptoms but also to prevent the establishment of myiasis-causing flies in areas where it is not endemic. The diagnosis mainly relies on the clinico-entomological criteria. The latter includes the identification of fly larvae in the infested tissues or organs. In the case of cavernous lesions, the extraction of larvae in a single session is difficult, and this delay may aggravate the situation. As long as larvae are present, a foul-smelling bloody discharge is observed [19]. Clues that myiasis may be present include recent travel to tropical and sub-tropical endemic regions, non-healing cutaneous lesions, discharge from a central punctum, movement sensation under the skin, pruritus, or pain [20]. Dermoscopy, imaging, ultrasound scanning, and serology are techniques used to diagnose myiasis [21,22,23,24]. The biopsy of the wound may also be advised for accurate microscopic examination. Blood tests, such as a complete blood count, may show increased white blood cells. IgE may also be elevated as another manifestation of the host–parasite interaction. In recent decades, molecular biology has been used in diagnosing myiasis in various countries [25].

The treatment of myiasis essentially consists of the manual removal of larvae, although the removal of larvae from tissue can be difficult due to the larva’s tapered shape and the many rows of spines and hooks that it uses to grip tissue (anterior hooks that function as an anchor). On the other hand, larvae require contact with air to breathe. The application of suffocating occlusive substances, such as Vaseline, glycerol, paraffin, petrolatum, or bacon, restricts oxygen flow and forces the larvae to move on the surface for respiration, consequently facilitating the removal from myiasis lesions [26]. Medications may also be prescribed in some cases of myiasis. Ivermectin is a semisynthetic macrolide antibiotic that is effective and can be prescribed for the treatment of more severe cases [27]. Surgery is typically performed to remove a severe infection such as migratory myiasis (deep migration into tissue in which extraction may not be possible) [28]. Proper hygienic cleaning methods are complementary preventive measures that should be strictly followed for wound cleaning after the removal of the larva.

*Cochliomyia hominivorax* is a parasitic fly belonging to the Calliphoridae family and *Cochliomyia* genus. The latter includes four species of *C. macellaria*, *C. hominivorax*, *C. aldrichi*, and *C. minima* [29]. *Cochliomyia hominivorax* is known as screwworm and is the main agent of myiasis in the New World [30,31,32,33]. It was first described by a French naval surgeon, Dr. Charles Coquerel, in 1858 when it was responsible for the death of hundreds of prisoners at the Devil’s Island penal colony in French Guiana [34]. It is an obligate ectoparasite in homeothermic vertebrates, either domestic or wild, occasionally including humans. Its larvae produce myiasis and primarily feed on living warm-blooded animals (such as cattle and other livestock). The larvae can cause ocular [35], oral [36], umbilical [37], subcutaneous [38], and nasal [39] infections. In addition, *C. hominivorax* is responsible for the most common forms of human myiasis [40] and causes serious lesions in the abdomen, lower limbs, and various parts of the head (ears and eyelids) [41]. This feeding of the larvae on the host causes deep, pocket-like lesions in the skin, which can damage infected tissues. However, *C. hominivorax* infections in humans are less common and occur in patients with the following risk factors: open wounds, poor hygiene, low educational level, alcoholism, immobility, and physical or mental disability [42]. The female flies lay eggs on a superficial wound, injured mucous membrane, or the natural orifice of a warm-blooded animal or humans (such as nostrils, sinuses, mouth, orbits, and genital orifices). One female can deposit up to 400 eggs at a time, and up to 2800 eggs during a 10–30 day lifespan [43]. Eggs hatch between 12 and 24 h into larvae that burrow into the wound or flesh to feed. Unlike typical maggots that feed on dead flesh, *C. hominivorax* larvae feed on living tissue that allows the larvae to penetrate the tissues [44]. Larvae are pointed at one end and blunt at the other, with dark brown spines circling the body [45]. They possess small spines on each body segment that resemble a screw’s threads. The third instar larvae of *C. hominivorax* are creamy-white with a robust cylindrical body from 6 to 17 mm long and 1.6 to 3.5 mm wide [46]. They penetrate the wound and burrow deeper, perpendicular to the skin surface. The larvae then continue to feed on the wound fluids and the tissue. The wound enlarges as the larvae feed, and a foul-smelling, bloody discharge develops. There may be hundreds of larvae within the wound. The wound produces a characteristic odor, often not perceived by humans, which attracts other gravid female flies to lay their eggs in the same wounds and aggravates the infective process [47]. After 4–10 days of feeding, larvae fall out on the ground, burrow into the soil, and then transform into pupa. The pupal stage lasts from a week to three months approximately [47]. The rate of development of the immature stages is influenced by environmental and wound temperatures and becomes slower at lower temperatures with no true diapause. The adult screwworm flies emerge and mate after 3–5 days, beginning the cycle again. Adult flies are less frequent than larvae and can be separated from flies of other genera by the confirmation of a deep blue to blue-green metallic body colour with three dark longitudinal stripes on the thorax.

The risk of the introduction of *C. hominivorax* may be relatively low, but it can be introduced and spread into non-endemic and eradicated areas via the movement of infested hosts, including humans. According to World Organization for Animal Health (WOAH), *C. hominivorax* is listed as a notifiable infestation for animals that affects livestock, wildlife, and humans in endemic areas and leads to major socioeconomic consequences [48]. Therefore, *C. hominovorax* infestation can be health-threatening, since it can penetrate living tissues of the body and does not stay subdermal, contrary to most myiasis-causing fly species. Extensive, chronic, or advanced-stage wounds with frequent exposures are commonly infested by this fly species [42,49]. Massive tissue destruction, the loss of eyes and ears, and erosion of bones and nasal sinuses can occur following the *C. hominovorax* infestation. Severe cases may be accompanied by fever, chills, pain, bleeding from infested sites, and secondary infections. Blood tests may show elevated levels of neutrophils and eosinophils [50]. It can be fatal in untreated severe infestations [51]. 

Beyond the sanitary problems associated with this species, these infestations affect veterinary sectors, such as the cattle industry. In animals, myiasis caused by *C. hominivorax* results in a negative economic impact due to a decrease in animal weight and milk production, and an increase in animal death. Infected animals may present with enlarged, draining, and foul-smelling wounds. They may isolate themselves and show signs of discomfort. Infected animals with no intervention may die from secondary infection or toxicity within 7–14 days. A control cost (e.g., cost of larvicides) of USD 2 m per year is estimated for eliminating animal myiasis [52].

Human myiasis caused by *C. hominivorax* continues to be reported in endemic countries, especially in Central and South America and some Caribbean islands [53,54]. The northern and southern borders of its range are primarily limited due to cold weather [55]. It has been controlled using sterile insect techniques since the 1950s. Millions of mass-produced sterile flies are released per week during a campaign. Eradication has been achieved in the U.S.A. (by 1982), Mexico (by 1991), and most other Central American countries. A permanent barrier was established at the Panama–Colombian border. In this barrier, sterile flies are continuously released to prevent reinfestation in South America [56]. Economic savings in the eradicated areas, spanning the southern US to Panama and parts of the Caribbean, is estimated to be USD 1.3 billion per year [47,57]. Furthermore, eradication has also been achieved in Curacao, the Netherlands Antilles, the British Virgin Islands, the USA Virgin Islands, and Puerto Rico. Nevertheless, human myiasis has been reported in individuals who have traveled to these countries [58]. Based on computer simulation models, *C. hominivorax* can potentially colonize most of the tropical and semi-tropical regions of the world [59,60]. International livestock trade and movement, as well as human travel, have resulted in the introduction of *C. hominivorax* to non-endemic areas and previously eradicated areas [61]. *Cochliomyia hominivorax* was introduced and established in Libya in 1988, [62] presumably by livestock importation from South America [63]. In a similar way, it entered France through an infected dog returning from Brazil [64]; and in Australia by a woman who visited Brazil and Argentina [65]. Despite the announcement of Mexico as a screwworm-free country in 1991, 69 cases were reported in 1992 and 1993, about 200 miles away from the USA border [66]. Similarly, several screwworm reintroductions have been reported in the USA since their complete eradication in the 1980s [60,67]. On the other hand, the control management strategies of these parasitic flies in South America rely mainly on the application of insecticides, which are harmful to the environment and make them less efficient due to the emergence of resistant populations [68]. Furthermore, it affects non-target organisms.

French Guiana is a French overseas department located on the Northeastern coast of South America in which some myiasis cases, including furunculoid and cavitary myiases, have already been reported [23,69]. Nevertheless, in spite of the developed knowledge of myiasis worldwide, little is known about this parasitosis and its causative agents in this tropical country, French Guiana. Herein, we present the case of an immunocompetent patient with long-standing wound myiasis.

## 2. Case Presentation

A 34-year-old man was consulted twice two weeks apart at the hospital of Cayenne, the main city of French Guiana, for otorrhagia and otalgia in the left ear. He complained of constant noise and tickling coming from the same ear. He was originally from Paris, France, and had lived for several years on Reunion Island, in the Indian Ocean, before moving to French Guiana less than a year earlier. The patient was an agricultural engineer, who worked for environmental protection. He worked mainly in the peri-urban area of Cayenne but regularly hiked in exceptional biogeographical areas, such as the primary forest. His residence was healthy with full access to amenities, located in a low urbanized area and surrounded directly by secondary forests and swamps. The patient presented no stigma of negligence and was aware of necessary local care, hygiene, and dietary rules. Except for smoking, no notion of diabetes mellitus, chronic alcoholism, or immunosuppression, including AIDS, was recorded. He had a history of multiple surgeries in the left ear, including the installation of transtympanic aerators in 1991, diagnosis and removal of cholesteatoma in 2003, 2005, 2008, and 2017, ossiculoplasty in 2008, and tympanoplasty in 2017. He underwent a surgical operation, the so-called open technique, in which a cavity is dug in the temporal bone that opens into the external auditory canal at the level of its posterior wall, forming a sort of cul-de-sac at the bottom of the external auditory canal. The patient suffered from sequential hypoacusis due to cholesteatoma surgery. In addition, he was subjected to occasional otorrhagia in the left ear since its arrival in French Guiana. In mid-December 2020, a strong analgesic drug was prescribed following the patient’s arrival at the emergency department for a painful otorrhagia, leading to hospitalization the next morning. 

**Figure 1:**
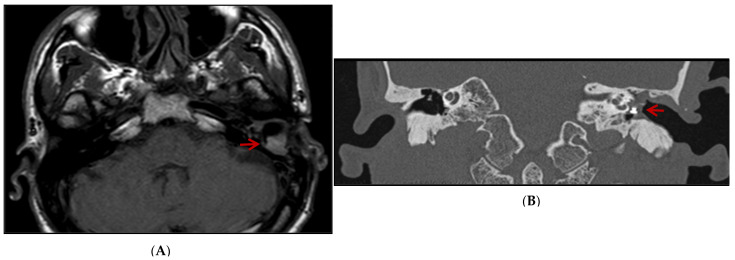
MRI axial (**A**) and CT scan coronal (**B**) images: scar lesion filling the left middle ear of the patient (highlighted by red arrow) compared to the right ear.

Based on clinical examination, an ear canal inflammation, along with myiases protruding from the external auditory canal, a non-marginal post-inferior perforation of the left eardrum, and a thick complete graft were observed. MRI demonstrated scarring lesions of the myiasis filling the middle of the ear space (Figure 1A). A CT scan showed the left middle ear filled with tissues enclosing the ossicular prosthesis (Figure 1B). He then underwent treatment, including fulfilling the ear cavity with topical ivermectin (5 crushed tablets of 3 mg) and Vaseline covered by an occlusive dressing for 48 h. An aspiration performed right after this intervention led to the isolation of several tens of maggots from the mastoid cavity. Patient care was continued on an outpatient basis with borated hydrogen peroxide ear baths twice a day for 7 days. Two weeks later, the patient felt an insect entering the left ear’s external auditory canal, crawling and making noise for 10 min without being able to pull it out. A bloody otorrhagia, otalgia, and scratching appeared 2 days after this event, leading to referring the patient again to the emergency department resulting in receiving the same treatment as two weeks ago. Following the observation of a teeming mass of maggots at the top of the external auditory canal close to the eardrum, they were aspirated the next day. No otorrhagia was observed after two days. The outcome was favorable within 3 months, and no relapse was reported by the patient. It is worth mentioning that the patient consulted for a dengue infection diagnosed by NS1 antigen detection a few days after the appearance of myiasis, without an obvious correlation between both medical events.

**Figure 2:**
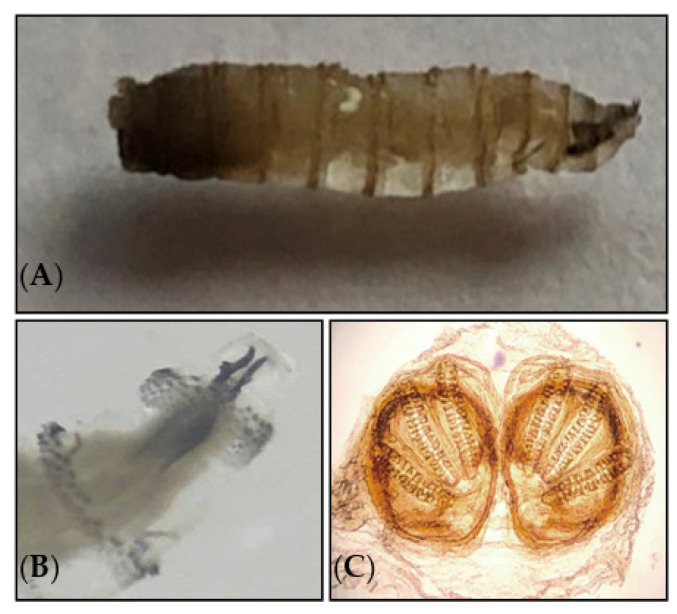
(**A**) Third-stage larva of *Cochliomyia hominivorax* (Calliphoridae). (**B**) Cephalopharyngeal skeleton. (**C**) Posterior spiracle of larva examined under a light microscope at 500× magnification.

In order to confirm the diagnosis and to identify the fly species, the larvae responsible for parasitosis were extracted by the patient himself, preserved in 70% alcohol, and sent to mainland France to the parasitology–mycology department of Avicenna Hospital (Bobigny, France) for an accurate identification. The morphological identification was performed based on the entomological criteria, following the dissection of the larvae and the identification of the cephalopharyngeal skeleton and posterior spiracles under the microscope as *Cochliomyia hominivorax* [1,70] (Figure 2, Supplementary information S1). 

**Figure 3:**
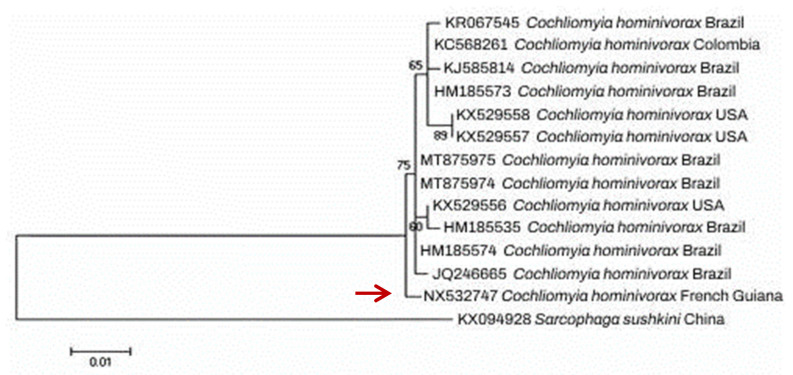
Neighbor-joining (NJ) tree reconstructed from COI sequence of *Cochliomyia hominivorax* specimen isolated from our patient (highlighted by red arrow) together with homologous sequences collected from GenBank.

Morphological identification was further confirmed using a molecular approach. For this purpose, the larvae DNA was extracted using Chelex 10% (Bio-Rad, Hercules, CA, USA) and then subjected to conventional PCR and bidirectional sequencing, targeting 710 bp fragment of the mitochondrial cytochrome oxidase I gene (mtCOI) [71]. BLAST analysis identified the specimen as *C. hominivorax* according to >99% identity with GenBank sequences. The obtained sequence was deposited in GenBank with the assigned accession number NX532747. An inferred neighbor-joining (NJ) phylogenetic tree of COI sequences belonging to our *C. hominivorax* specimen (AVC1) and homologous GenBank sequences, constructed using MEGA version 5.0 software, demonstrated a high genetic diversity and significant heterogeneity compared to homonym sequences of other countries, such as from North and South America (Figure 3).

## 3. Discussion

French Guiana is a coastal department surrounded by Brazil, Suriname, and the Atlantic Ocean, in which more than 90% of its surface is covered by Amazonian rainforest. It possesses a tropical, hot, and humid climate throughout the year, with a relatively dry, slightly warmer season from July to November, and a rainy season from December to June. Due to the abundance of natural resources and rainfall, it is rich in insect species in terms of frequency and biodiversity.

The accurate diagnosis of myiasis infestation relies mainly on the medical background, geographic location, travel history, and clinical examination of an infected patient. However, some complications related to myiasis can occur. These include allergies (due to not completely removing the larvae), secondary pyogenic infection, cellulitis, pneumocephalus, extensive erosion of the tissue or mucous membranes, meningitis, and death (in unattended and untreated cases) [72]. In addition, the presence of larvae in the tissue triggers a local inflammatory response with the migration and proliferation of inflammatory cells, such as neutrophils, mast cells, eosinophils, fibroblasts, and endothelial cells [73]. Furthermore, a complete blood cell count may show high levels of leukocytes and eosinophils [74]. In the case of our patient, an ear canal inflammation along with a painful otorrhagia were the two major complications recorded. 

On the other hand, the correct identification of the larvae species is essential for understanding the infestation mechanism and promoting preventive and treatment measures [54,75]. The morphological identification of the larvae relies mainly on the body shape, the mouthparts, posterior spiracles, and the arrangement of cuticular spines [76]. The posterior spiracles are usually visualized by dissection under a stereomicroscope, which allows identifying the fly species responsible for myiasis at the species level. In the present study, the larvae were identified according to the general appearance of the larva (morphological criteria, such as color and size) and examination of the posterior respiratory spiracles as *C. hominivorax* (Figure 2). In order to confirm morphological identification and to investigate the genetic diversity of fly larva species isolated in this study with counterpart sequences from other endemic countries, a conventional PCR targeting COI fragment was performed that allowed us to further confirm the identity of the larva species. Furthermore, high heterogeneity was observed between the larval specimen and GenBank sequences (Figure 3). 

To the best of our knowledge, seven myiasis reports have been documented in French Guiana. It includes three reports of cutaneous myiasis [77,78,79], followed by two reports of furuncular myiasis [80,81], and one report of nosocomial myiasis [82]. Most of the infected patients were males, with inflammatory nodules as the most abundant symptom exhibited. *Dermatobia hominis* was the most frequently reported fly species responsible for myiasis in this country. A single report of myiasis caused by *C. hominivorax* was reported (nosocomial myiasis) in a bed-ridden 84 years old woman, who developed right nasal myiasis during his stay at the Cayenne Hospital [82]. In contrast to the previous report, our patient was an immunocompetent young patient with no history of recent travel to other endemic regions where *C. hominivorax* is prevalent. This report is, therefore, the second case of myiasis caused by *C. hominivorax* and the first case of wound myiasis in this country. Detailed information on the myiasis cases reported in French Guiana is provided in Table 1. The information gathered in Table 1 is of significant importance not only for the inhabitants but also for the tourists of the Amazonian Forest regarding increasing travel to tropical regions. 

Various treatments have been proposed for the treatment of myiasis, but most of them are not achievable in outpatient consultation. The management of myiasis usually consists of (i) mechanical removal of the larvae, (ii) surgical debridement of the infested wound tissue, and (iii) irrigation of the wound with antiseptic solutions [83,84]. In addition, topical medications such as ivermectin have been successfully used for the removal of larvae [85,86]. The application of topical ivermectin combined with Vaseline and manual extraction of the maggots was effective in eradicating the ectoparasites with no need for surgery. A favorable evolution and infection cure were observed two weeks post-treatment. The prevention of reinfestation and the spread of larvae are important underlying conditions in the treatment of the disease. This dreadful condition can be prevented by the maintenance of appropriate hygiene, general cleanliness, sanitation, proper wound cleaning, decreasing the number of flies around the wounds, and educating the population susceptible to the disease. In the case of the patient presented in this study, he had no underlying medical condition. However, a large cavity left by several ear surgeries provided a suitable environment for this myiasis colonization. 

Wearing protective clothing, using insect repellant, covering wounds, and improving general sanitation and personal hygiene are preventative measures to avoid myiasis [56]. In non-endemic areas, the surveillance of all imported animals is necessary to prevent the entry of screwworms. Despite several cases of negligence or lack of hygienic measures associated with myiasis frequently reported in the literature, this patient had a local debilitating condition (a multi-scarring aspect of his left auditory canal) without any negligence and was aware of the local care of his infested ear. Nevertheless, he was infested during his daily life activities in the vicinity of Cayenne, the capital, and not in the deep forest or rural areas. This case highlights the risk of myiasis infestation outside the known and usual disease zones and the presence of this pathogenic ectoparasite near urban areas.

## 4. Conclusions

The entomological identification of the ectoparasite and the description of this case are important to raise the knowledge on the Amazonian ectoparasites, which are too often noted and rarely studied. With the increasing number of international travelers to the Amazonian tropical regions, it is important to acquire knowledge on this parasitosis, especially for healthcare workers unfamiliar with myiasis and its etiological agents. Therefore, general practitioners and surgeons in these regions should be better informed about the risk of ectoparasitic colonization in their patients without underlying comorbidities, but with the sequelae of cavities.

## Figures and Tables

**Table 1:** Detailed information on the myiasis cases reported in French Guiana.

	Patient			Etiologic Species	Infected Location	Clinical Symptom	Treatment	Reference
Myiasis Type	Age (Years Old)	Sex	Myiasis Origin					
Cutaneous myiasis	46	♂	French Guiana	*Dermatobia hominis*	Scalp	Scalp inflamation and impetigo	Incision	[77]
Furuncular myiasis	?	?	French Guiana	?	Eye	?	?	[69]
Ophthalmomyiasis (9 cases)	1.5 months to 45	♂♀	8 (French Guiana) and 1 (Brazil)	*Dermatobia hominis*	Eye	Varied depending on the patient’s case	Petroleum ointment, ivermectin	[82]
Nosocomial myiasis	84	♂	French Guiana	*Cochliomyia hominivorax*	Right nasal	Posterior purulent rhinorrhoea and edema on the forehead	Oral ivermectin	[81]
Cutaneous myiasis	31	♂	French Guiana	*Dermatobia hominis*	Limbs	Inflammatory nodule	Topical disinfectants and systemic amoxicillin/clavulanic acid	[78]
Cutaneous myiasis	66	♂	French Guiana	*Dermatobia hominis*	Forearms	Inflammatory nodule	pressing the coil, paraffin oil	[79]
Furuncular myiasis (3 cases)	39 ^1^, 42 ^2^, 59 ^3^	♂ ^1^, ♀ ^2^, ♀ ^3^	French guiana ^1^, Senegal ^2^, Cameroon ^3^	*Dermatobia hominis* ^1^, *Cordylobia anthropophaga* ^2^, *C. rodhaini* ^3^	Leg ^1^, left buttock ^2^, scalp & arm ^3^	Punctum ^1^, inflammatory nodule ^2^, scalp nodules, pain, fatigue, and facial edema ^3^	Varied depending on fly species	[80]

Superscripted numbers of 1, 2 and 3: indicating clinico-epidemiological criteria of three patients reported in [80].

## Data Availability

Not applicable.

## Supplementary Materials

The following supporting information can be downloaded at: https://www.mdpi.com/article/10.3390/diagnostics13152575/s1. Supplementary information S1: Third-stage larva of *Cochliomyia hominivorax*.
