# Supplementary figures and images for: Morphological and Molecular Characterizations of Cochliomyia hominivorax (Diptera: Calliphoridae) Larvae Responsible for Wound Myiasis in French Guiana
Source: Diagnostics (Basel). 2023 Aug 2;13(15):2575. doi: 10.3390/diagnostics13152575 (PMC10416906; doi:10.3390/diagnostics13152575)

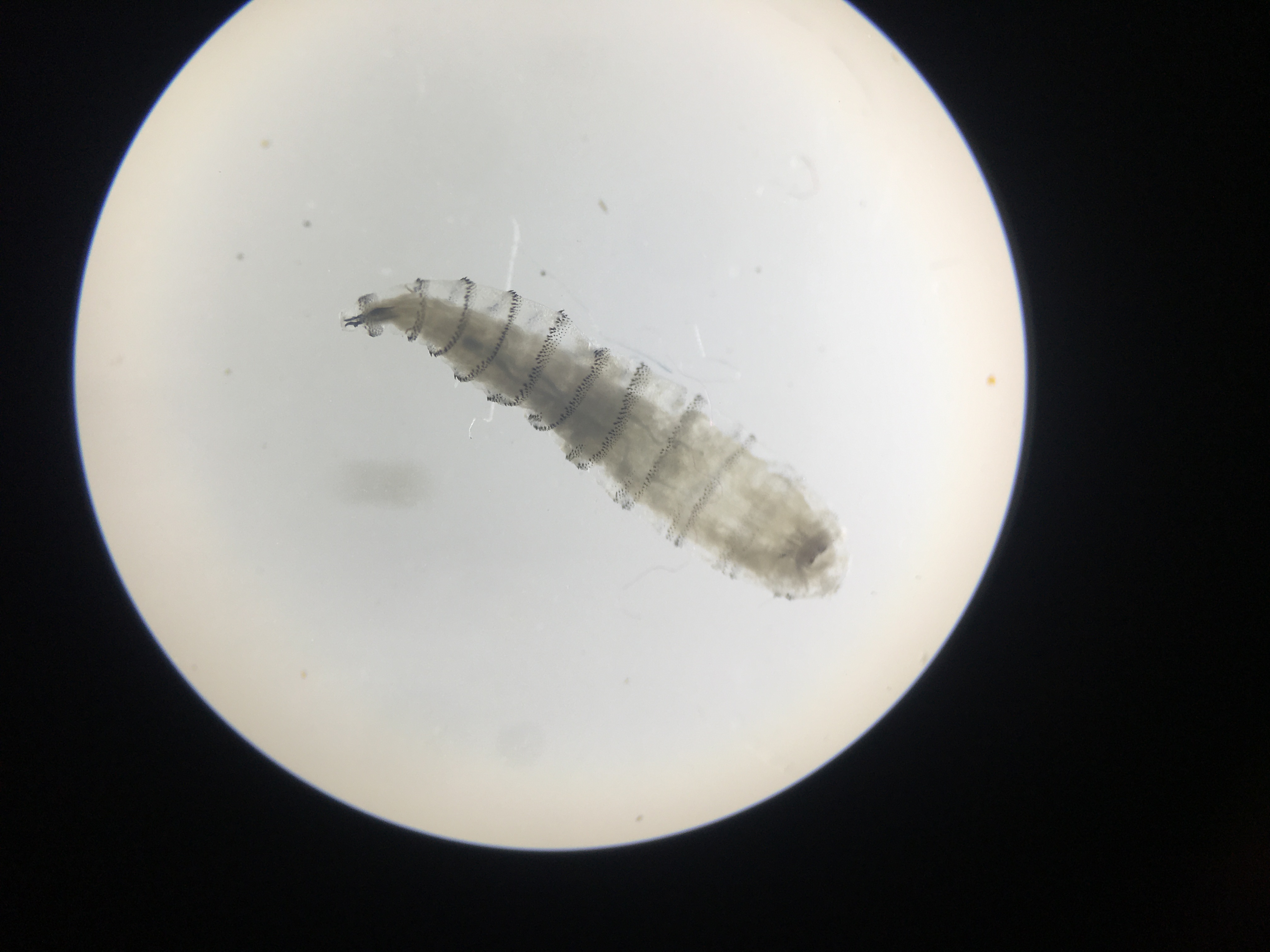

Supplement: Supplementary file 1 [file diagnostics-13-02575-s001.zip › C hominivorax (1).JPG]

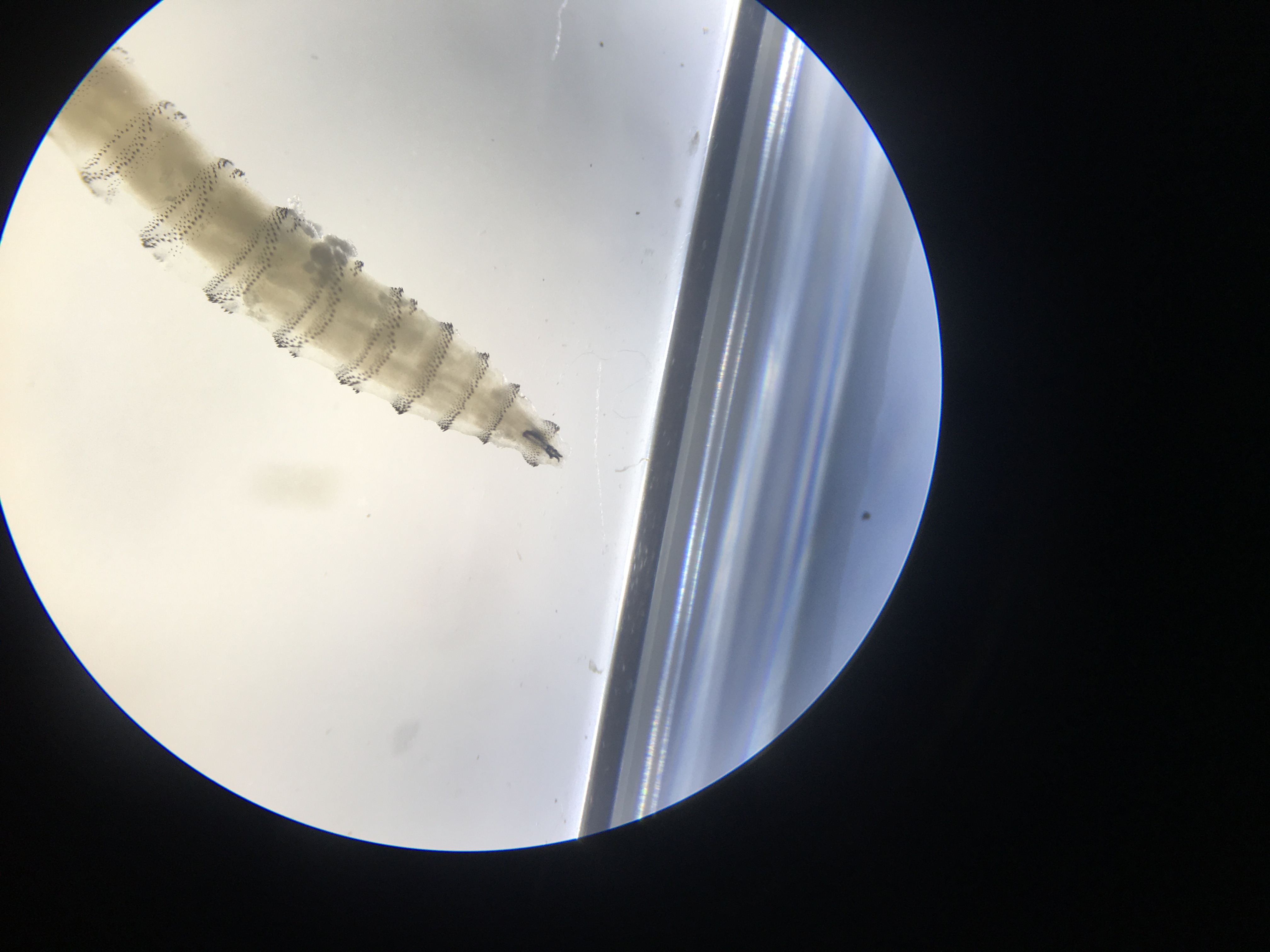

Supplement: Supplementary file 1 [file diagnostics-13-02575-s001.zip › C hominivorax (2).JPG]

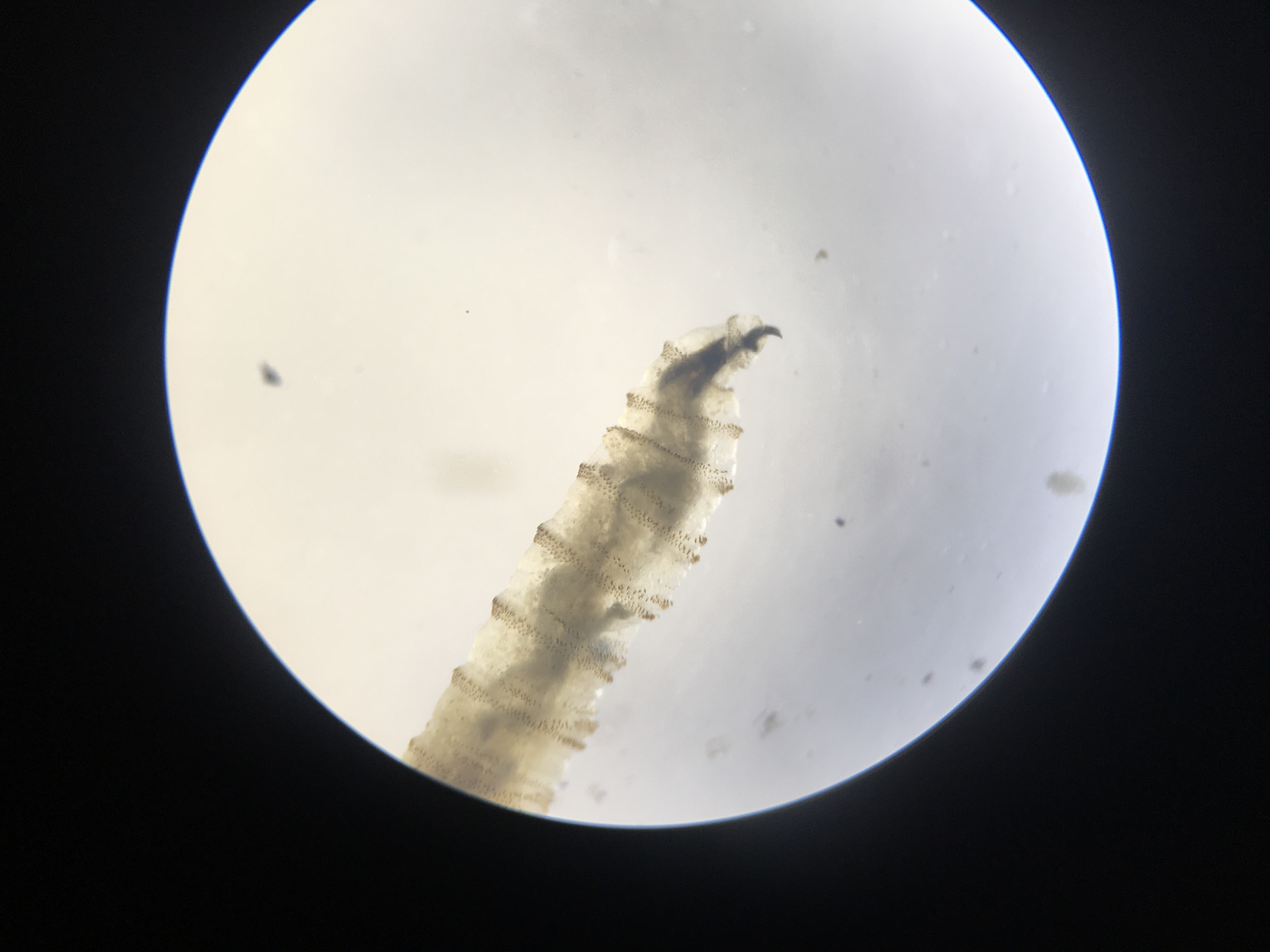

Supplement: Supplementary file 1 [file diagnostics-13-02575-s001.zip › C hominivorax (3).JPG]

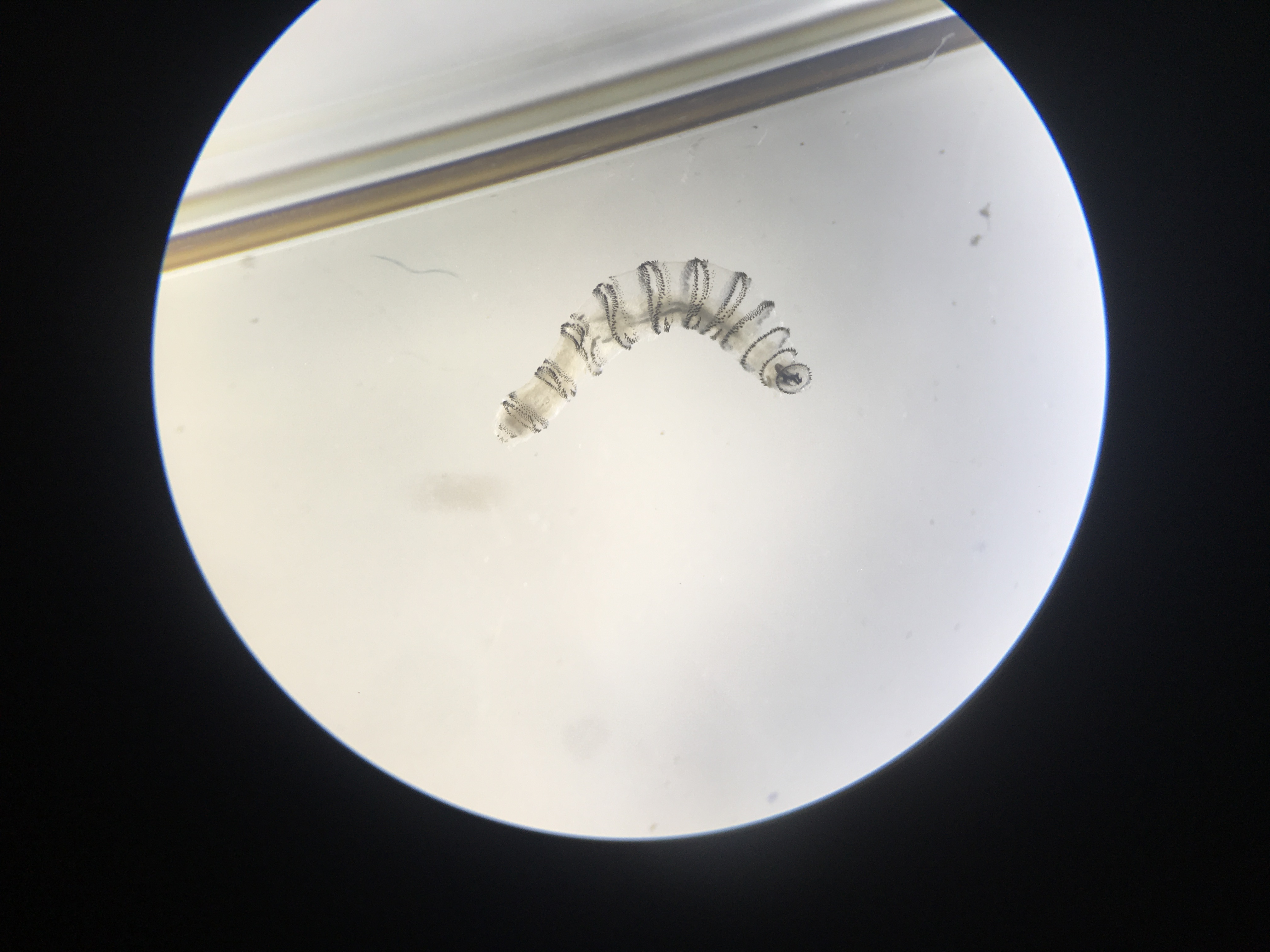

Supplement: Supplementary file 1 [file diagnostics-13-02575-s001.zip › C hominivorax (4).JPG]
